# The Evolutionary Panorama of Organ-Specifically Expressed or Repressed Orthologous Genes in Nine Vertebrate Species

**DOI:** 10.1371/journal.pone.0116872

**Published:** 2015-02-13

**Authors:** Libing Shen, Gangbiao Liu, Yangyun Zou, Zhan Zhou, Zhixi Su, Xun Gu

**Affiliations:** 1 State Key Laboratory of Genetic Engineering and MOE Key Laboratory of Contemporary Anthropology, School of Life Sciences, Fudan University, Shanghai, PR China; 2 Department of Genetics, Development, and Cell Biology, Iowa State University, Ames, Iowa, United States of America; University of California, Los Angeles, UNITED STATES

## Abstract

RNA sequencing (RNA-Seq) technology provides the detailed transcriptomic information for a biological sample. Using the RNA-Seq data of six organs from nine vertebrate species, we identified a number of organ-specifically expressed or repressed orthologous genes whose expression patterns are mostly conserved across nine species. Our analyses show the following results: (i) About 80% of these genes have a chordate or more ancient origin and more than half of them are the legacy of one or multiple rounds of large-scale gene duplication events. (ii) Their evolutionary rates are shaped by the organ in which they are expressed or repressed, e.g. the genes specially expressed in testis and liver generally evolve more than twice as fast as the ones specially expressed in brain and cerebellum. The organ-specific transcription factors were discriminated from these genes. The ChIP-seq data from the ENCODE project also revealed the transcription-related factors that might be involved in regulating human organ-specifically expressed or repressed genes. Some of them are shared by all six human organs. The comparison of ENCODE data with mouse/chicken ChIP-seq data proposes that organ-specifically expressed or repressed orthologous genes are regulated in various combinatorial fashions in different species, although their expression features are conserved among these species. We found that the duplication events in some gene families might help explain the quick organ/tissue divergence in vertebrate lineage. The phylogenetic analysis of testis-specifically expressed genes suggests that some of them are prone to develop new functions for other organs/tissues.

## Introduction

Metazoan adults, except sponges and *Placozoa*, consist of tissues and organs which are cellular organizations performing specific physiological functions. As one of major metazoan subgroups, vertebrates have a common body plan featured with a spinal column. Besides the spinal column, vertebrates share other conserved anatomical features such as heart and brain. These organs carry out the body functions which are crucial for the survival and reproduction of vertebrates. Although many vertebrate genomes have been sequenced and annotated, the details of functional genes or gene sets underlying each vertebrate organ largely remain unknown.

Invertebrate chordates such as sea squirt only have a rudimentary body system without kidney-like metanephridial organs. Its central nervous system is also primitive and contains only a neural tube instead of a well-developed brain. Nevertheless, lamprey, a basal vertebrate, possesses a more sophisticated body system which was inherited by all vertebrates. As far as the genome is concerned, the major difference between invertebrate chordates and vertebrates is that the vertebrate common ancestor experienced two rounds of whole-genome duplication while the basal chordates didn’t [1–3]. The large-scale gene duplication events inevitably led to either neofunctionaliztation or subfunctionalization of duplicate genes which in turn increased the organismal complexity [4–6], but how these events contribute to tissue and organ divergence is unclear.

Until recently, the advent of RNA-Seq technology has made the whole-genome expression data from a single biological sample much more accessible [7]. It is superior to the traditional microarray approach in terms of coverage, precision and sensitivity [8–10]. With the help of RNA-Seq technology, the transcriptome of an organ can be analyzed with unprecedented accuracy. A lot of efforts have been made to understand the alternative splicing profiles of different organs from different species [11, 12]. However, the study of the expression profiles underlying different organs from different species is somewhat stagnant.

The lately published RNA-Seq data including six organs from ten vertebrate species could provide some answers, at least from the gene-expression perspective, to the questions above [13]. By utilizing these RNA-Seq data and relevant whole-genome sequence data, we conducted an extensive analysis on the transcriptomes of six organs across nine vertebrate species. We found a number of organ-specifically expressed or repressed orthologous genes whose expression patterns are largely conserved among these species. We also investigated their evolutionary features and the transcription factors which might be involved in the control of these genes. Our study provides some insights into the evolution of vertebrate organs from a transcriptomic perspective.

## Materials and Methods

### RNA-Seq data

The RNA-Seq data of six organs (brain, cerebellum, heart, kidney, liver and testis) from ten vertebrate species (chicken, platypus, opossum, mouse, macaque, orangutan, gorilla, bonobo, chimpanzee and human) were downloaded from the supplementary information of Brawand et al. [13]. We calculated the RPKM (Reads Per Kilobase per Million mapped reads) value for each gene based on the downloaded data (unique read coverage per exon). Due to the uneven number of samples in some organs from some species, we used the mean RPKM value if multiple RPKM values were available for each organ per species. Except testis (orangutan testis data were not available), orangutan cerebellum (orangutan male cerebellum data were not available) and human liver (human female liver data were not available), each organ per species had two batches of RPKM values: one from female and the other from male. We transformed the RPKM values into the log_2_(RPKM) values and then calculated the Z-score for every log_2_(RPKM) value within each organ of each species, in order to render the gene expression values comparable among different organs and different species.

### Orthologous gene cluster

The orthologous gene information between chicken and platypus, opossum, mouse, macaque, gorilla, orangutan, chimpanzee, human was downloaded from Ensembl website (release 73). We didn’t include bonobo in our study, because bonobo is a subspecies of chimpanzee and Brawand et al. used the chimpanzee genome for mapping both bonobo and chimpanzee RNAseq data [13]. The number of orthologous genes between chicken and platypus, opossum, mouse, macaque, gorilla, orangutan, chimpanzee, human is 16676 (12952 with high orthology confidence|3724 with low orthology confidence), 15867 (13137|2730), 16764 (13608|3156), 17,092 (13425|3667), 16449 (12992|3457), 16108 (12645|3463), 15363 (12114|3249), and 16159 (12927|3232), respectively. We only used the genes with high orthology confidence for building orthologous gene cluster. By doing so, we excluded the genes which might be out-paralogs but not real orthologs. Low orthology confidence means that it is not clear whether these genes are real orthologs or not, but they are the best available orthologous gene candidates under the given data (please see Ensembl website for more technical details).

Using chicken orthologous genes as the orthology template (chicken inparalogs had been already clustered together), we organized the orthologous genes from nine species into orthologous gene clusters. The cluster containing at least one orthologous gene from each of nine species would be kept for further study. Within-species paralogous genes (created by within-species gene duplication events) are allowed in one cluster. We had total 7679 orthologous clusters and 5030 of them are one-to-one orthologous cluster (each species has only one representative gene in a one-to-one cluster).

### Identification of organ-specifically expressed or repressed othologous gene clusters

First, one-way ANOVA test was employed to compare the mean expression Z-scores between any two organs in an orthologous gene cluster. Tukey’s test was used for multiple pairwise comparisons among six organs. There are 15 ANOVA results per cluster. For one cluster, if the ANOVA results showed that its expression level in one organ is significantly higher or lower than its expression levels in the rest ones, it would be taken as organ-specifically expressed or repressed (OSER) orthologous gene candidate cluster.

Second, we used their expression values (RPKM values) to examine these candidate clusters. If all genes in a candidate cluster averagely express at least 50% higher or lower in one organ than the rest ones, this cluster is then regarded as OSER orthologous gene cluster and the genes in OSER clusters are considered OSER genes. By doing so, we could eliminate the genes with only slight increase or decrease of expression in a specific organ, which are unable to be detected by ANOVA test alone.

The R package version 3.0.2 was used to perform statistical analyses in this study and a p-value smaller than 0.05 was viewed as statistically significant.

### Functional enrichment analysis

We used DAVID Bioinformatics Resources to perform gene-GO term enrichment analysis for OSER genes [14, 15].

### Gene family construction

Gene families were built from the orthologous and paralogous gene information downloaded from Ensembl website (release 73). Ensembl used all-against-all Blast e-values (statistical threshold) to cluster genes into evolutionarily related groups which include both orthologous and paralogous genes [16]. The species used for gene family construction also include anole lizard, clawed frog, zebrafish, lamprey and sea squirt. Although we don’t have the expression data for these five species, their genome data could facilitate our phylogenetic and evolutionary analyses.

### Sequence alignment, phylogenetic analysis and estimation of evolutionary rates

The whole genome protein sequences of 14 species were downloaded from Ensembl database. The longest transcripts of each genome were extracted with a Perl script. All protein sequences in each gene family were aligned using MUSCLE software with default parameters [17]. FastTree was used to construct phylogenetic trees for both gene families and one-to-one orthologous clusters with WAG model and "gamma" likelihood [18]. We used WAG model and "gamma" likelihood for branch length estimation, because WAG model is a general empirical model of protein evolution which is suitable for many gene families while "gamma" likelihood assumes that different parts of a gene could evolve at different rates [19].

We estimated the evolutionary rates of OSER genes using only one-to-one cluster. A phylogenetic tree was constructed for each one-to-one orthologous OSER cluster. Then we summed the total branch length of each tree. FastTree uses the empirical amino-acid substitution matrix such as JTT or WAG to calculate the branch length, so the total branch length of a tree actually represents the cumulative evolutionary rate of all genes in the tree. A one-to-one cluster contains only nine genes (one gene per species) while a non-one-to-one cluster contains more than nine genes (perhaps several genes per species). Since we used the total branch length of phylogenetic tree to estimate the evolutionary rates of OSER genes, more genes means longer branch length which in turn distorts our estimation.

### ChIP-seq data

The ChIP-seq data of 119 human transcription-related factors were extracted from Gerstein et al. [20]. The proximal and distal binding information of these transcription-related factors was used to find out whether they might be involved in the regulation of human OSER genes or not. Mouse and chicken ChIP-seq data were downloaded from ChIPBase [21]. We used the ENCODE criterion (2.5kb form the nearest gene) to sort mouse and chicken transcription factors into proximal binding ones and distal binding ones.

## Results

### Organ-specifically expressed or repressed orthologous gene clusters

Through pairwise comparisons using ANOVA test and their expression values, we discovered a number of orthologous gene clusters that are specifically expressed or repressed in brain, cerebellum, heart, kidney, liver, and testis. Because brain and cerebellum are the main parts of central nervous system, their physiological functions are partly overlapped. Thus, we also searched for the clusters specifically expressed or repressed in nervous tissues (brain and cerebellum), which show no significant expression difference between brain and cerebellum while have a distinct expression pattern between nervous tissues and the other organs.

We identified 1521 OSER clusters out of total 7679 orthologous clusters. 88 OSER clusters are specially expressed in one organ and repressed in another at the same time (supporting information files). For example, MAPT (microtubule-associated protein tau) cluster is specially expressed in nervous tissues (brain and cerebellum) while it is specially repressed in liver. It proposes that MAPT gene is essential for the functions of nervous tissues, but is unwanted for the functions of liver. The number of OSER clusters in each organ/tissue is shown in Table 1. According to the number of OSER clusters, testis has the widest expression spectrum among six organs.

**Table 1:** The number of specifically expressed or repressed (OSER) orthologous clusters in each organ/tissue.

Organ/Tissue	No. of specifically expressed clusters	No. of specifically repressed clusters
Brain	98	9
Cerebellum	49	15
Heart	139	44
Kidney	130	2
Liver	140	154
Testis	453	46
Nervous tissue*	269	52

*The OSER clusters in nervous tissue show no significant expression difference between brain and cerebellum while have a distinct expression pattern between nervous tissues and the other organs.

### DAVID analyses of OSER genes

In order to verify the possible functions of these OSER genes, their gene-GO term enrichment analyses were performed using DAVID functional annotation software [14, 15]. For the OSER genes in each organ, the DAVID result shows that their biological process, cellular component, molecular function and KEGG pathway are congruent with the organ’s physiological functions (S1–S9 Tables). Most Benjamini-corrected false discovery rates in our DAVID analyses are statistically meaningful, although there is no statistically significant false discovery rate found with cerebellum-specifically expressed genes.

### The evolutionary origins of OSER clusters

We grouped OSER genes into different gene families. All 1521 OSER clusters come from 1187 gene families. The species used for gene family construction include anole lizard, clawed frog, zebrafish, lamprey and sea squirt. Therefore, we are able to trace the evolutionary origin of each OSER cluster. The evolutionary origins of the OSER clusters in each organ/tissue are shown in Table 2. The result indicates that newly emerged genes had been not specifically expressed or repressed in heart or kidney since the appearance of Amniota (lizard or chicken), while nervous tissues, liver and testis constantly integrate newly emerged genes to their expression spectra during the process of evolution.

**Table 2:** The evolutionary origins of OSER clusters in each organ/tissue.

Origin	Brain	Cerebellum	Nervous tissue	Heart	Kidney	Liver	Testis
Chordata (sea squirt)	86	54	250	134	112	232	404
Craniata (lamprey)	13	7	43	29	12	30	46
Gnathostomata (zebrafish)	4	3	20	19	6	25	37
Tetrapoda (clawed frog)	3	0	4	1	2	5	3
Amniota (lizard or chicken)	1	0	4	0	0	2	9
Total	107	64	321	183	132	294	499

### Distribution of the number orthologous clusters in the gene families containing OSER genes

**Figure 1:**
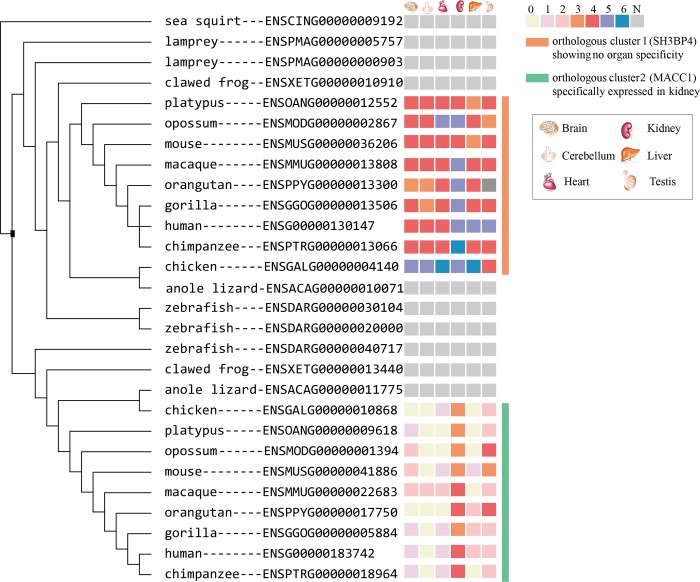
Phylogenetic tree of MACC1 gene family. A *Ciona intestinalis* gene was selected as the outgroup to root the tree and only the cladogram is shown. The tree node where a possible large-scale duplication event happened is marked with a filled black square ■. 7 means the gene’s expression level is higher than 95% of all genes expressed in the organ. 6 is between 95% and 85%. 5 is between 85% and 65%. 4 is between 65% and 35%. 3 is between 35% and 15%. 2 is between 15% and 5%. 1 is lower than 5%. 0 means no expression at all. N is not available.

The orthologous cluster in this study represents a group of genes which are derived from the same ancestral sequence and separated by speciation events. The homologous relationship between any two orthologous clusters within one gene family is paralogous, which means that these two orthologous clusters were created by a large-scale gene duplication event (Fig. 1). Therefore, the gene family containing more than one orthologous cluster means that it experienced at least one large-scale gene duplication event during its evolution.

**Figure 2:**
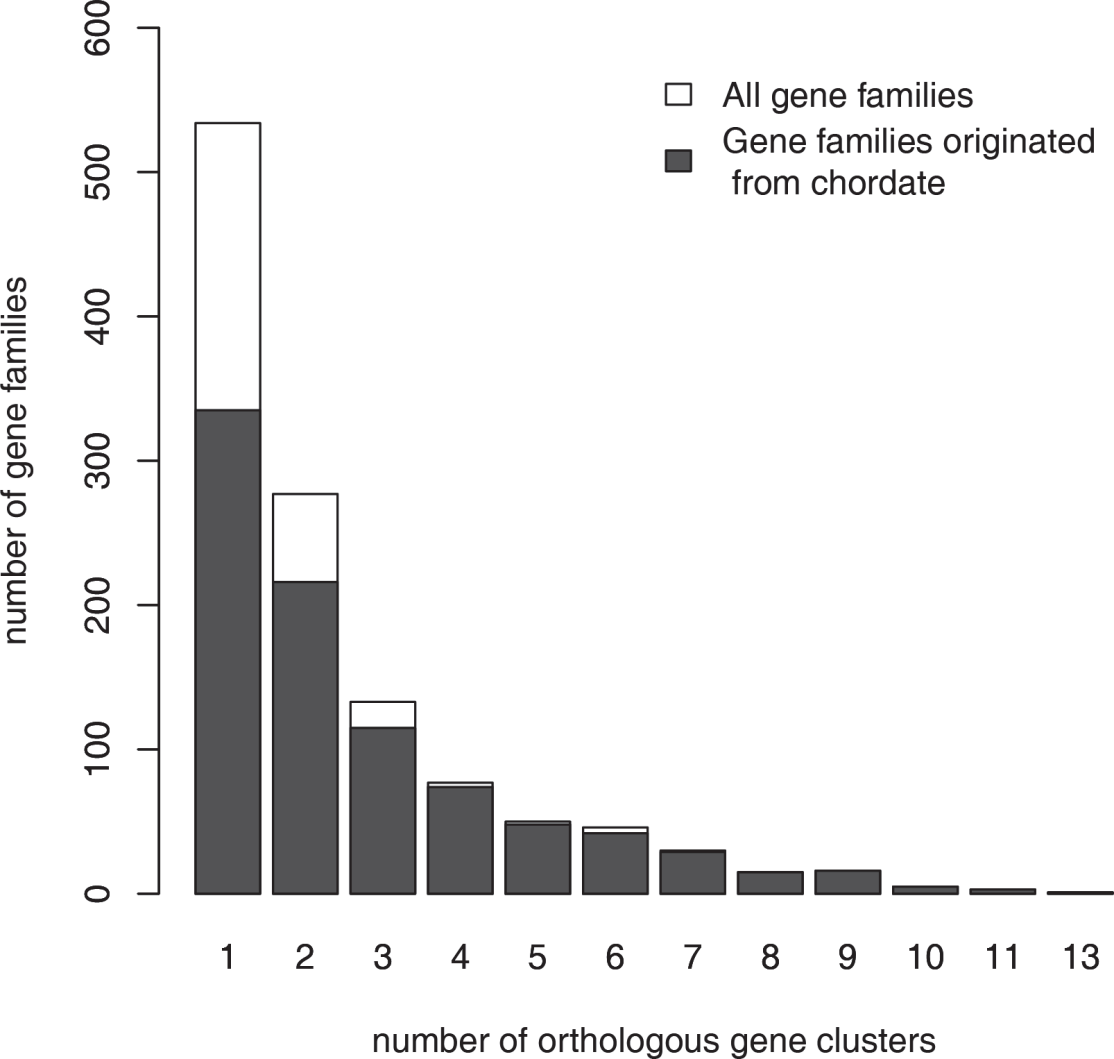
Distribution of the number orthologous clusters in the gene families containing OSER genes.

**Figure 3:**
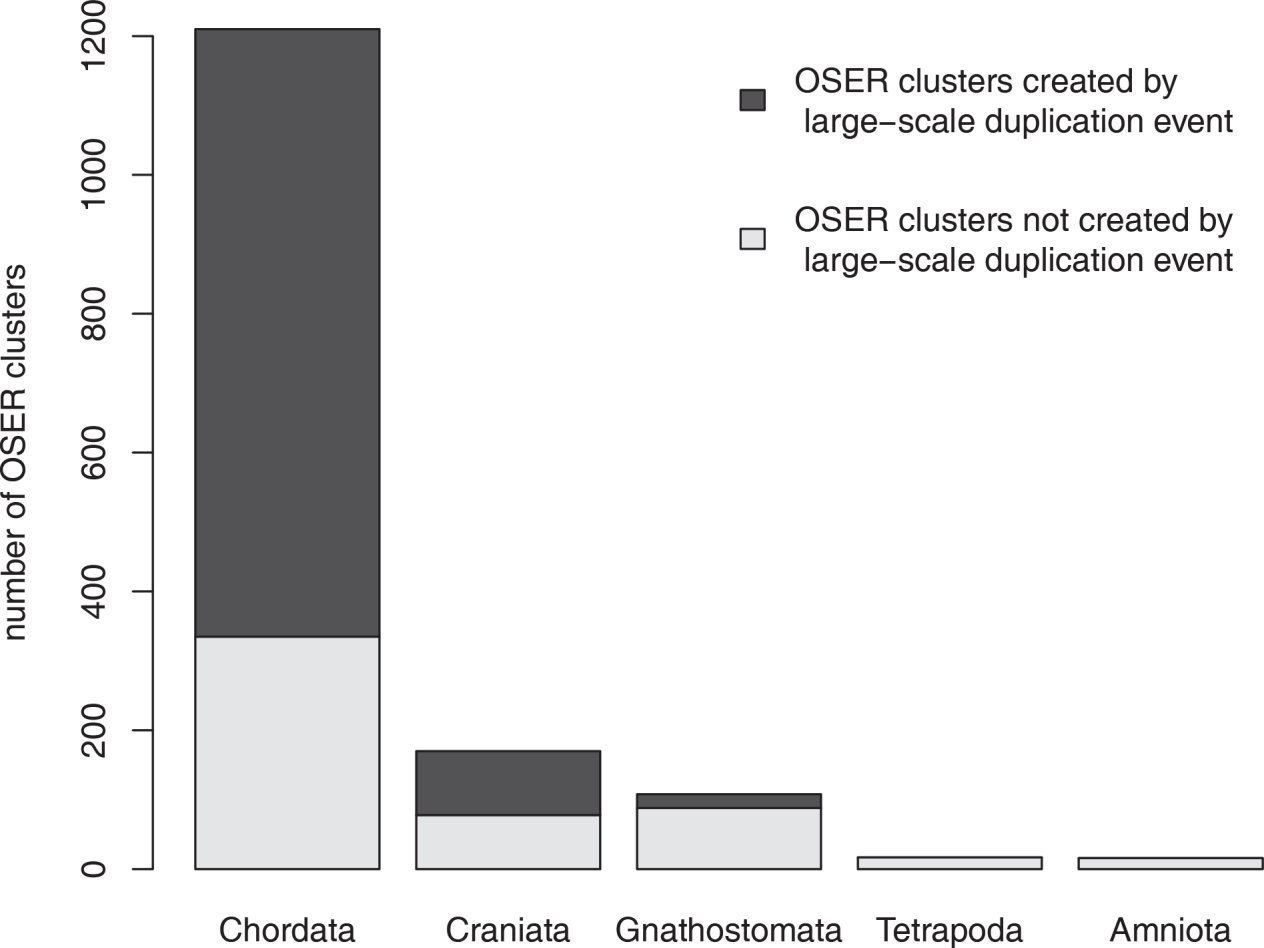
Classification of OSER clusters according to their evolutionary origins.

There are total 1187 gene families containing OSER genes and 899 of them can be traced to sea squirt. The distribution of the number orthologous clusters in these gene families is shown in Fig. 2. More than half of the families (653/1187) have more than one orthologous cluster. Consequently, 987 OSER clusters in these families are the result of at least one large-scale gene duplication event (Fig. 3).

According to their expression diversity, we classified all gene families into two categories: 1) the family contains one or more OSER clusters from the same organ/tissue; 2) the family contains the OSER clusters from two or more than two different organs/tissues.

Among 1187 gene families, 1016 families contain one or several OSER clusters from the same organ/tissue; 171 families contain the OSER clusters from two or more than two different organs/tissues. We calculated the average number of orthologous clusters in both categories. In the first category, the average number of orthologous clusters in each family is 1.99. In the second category, the average number of orthologous clusters in each family is 5.06. Wilcoxon test shows that the number of orthologous clusters are significantly different between these two categories (p-value < 2.2e-16).

Using the Z-score transformed data, we also examined the expression profiles of 1016 gene families in the first category. We divided them into two subcategories: 1) the expression profile of the gene family is the same as its OESR cluster(s); 2) the expression profile of the gene family is different from its OESR cluster(s). 513 families belong to the first subcategory and 503 families belong to the second subcategory. We also calculated the average number of orthologous clusters in two subcategories. In the first subcategory, the average number of orthologous clusters in each family is 1.52. In the second subcategory, the average number of orthologous clusters in each family is 2.48. The number of orthologous clusters of the first subcategory is significantly smaller than the one of the second subcategory (wilcoxon test, p-value < 2.2e-16). The results above show that the expression diversity of a gene family is correlated with the number of large-scale duplication events it experienced.

### The evolutionary rates of OSER genes

**Figure 4:**
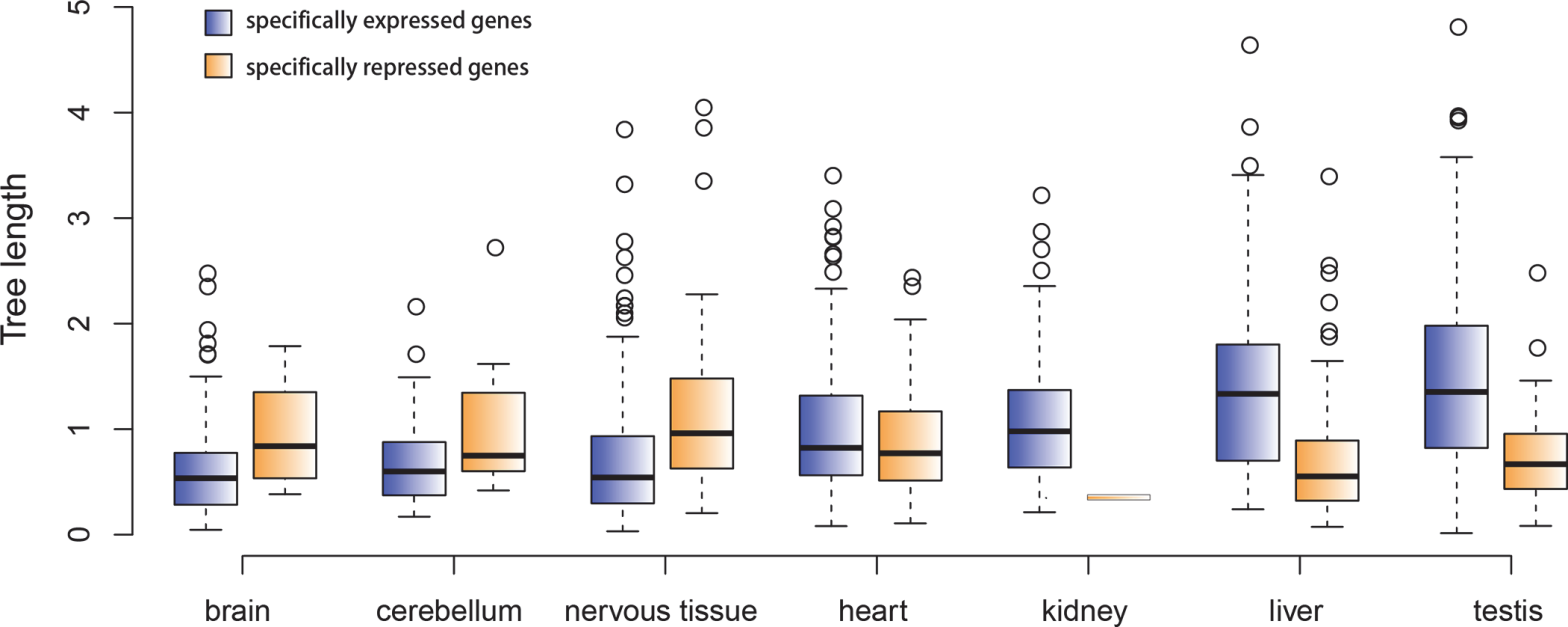
Comparison of the evolutionary rate of specifically expressed and repressed clusters in seven organs/tissues. The total phylogenetic tree branch length of each one-to-one OSER cluster was used to represent its evolutionary rate.

Multiple factors influence a gene’s evolutionary rate and two of major factors are expression spectrum and expression level [22]. We classified OSER genes into different groups based on their organ/tissue specificity and their expression or repression status in the organ/tissue. The average evolutionary rate of each OSER gene group is shown in Fig. 4. The genes specifically expressed in testis and liver have the highest average evolutionary rate while the ones specifically expressed in nervous tissues have the lowest average rate. The genes specifically expressed in heart and kidney have the intermediate average evolutionary rate.

In the same organ/tissue, the specifically expressed genes usually evolve faster than the repressed ones. However, this phenomenon is reversed in nervous tissues. The genes specifically repressed in nervous tissue have the highest average evolutionary rate among the repressed groups. They evolve significantly faster than the brain/cerebellum/nervous-tissue-specifically expressed genes (wilcoxon test, p-value < 1.9e-05).

### Organ-specifically expressed or repressed transcription factors and the transcription-related factors might be involved in regulating OSER genes

**Figure 5:**
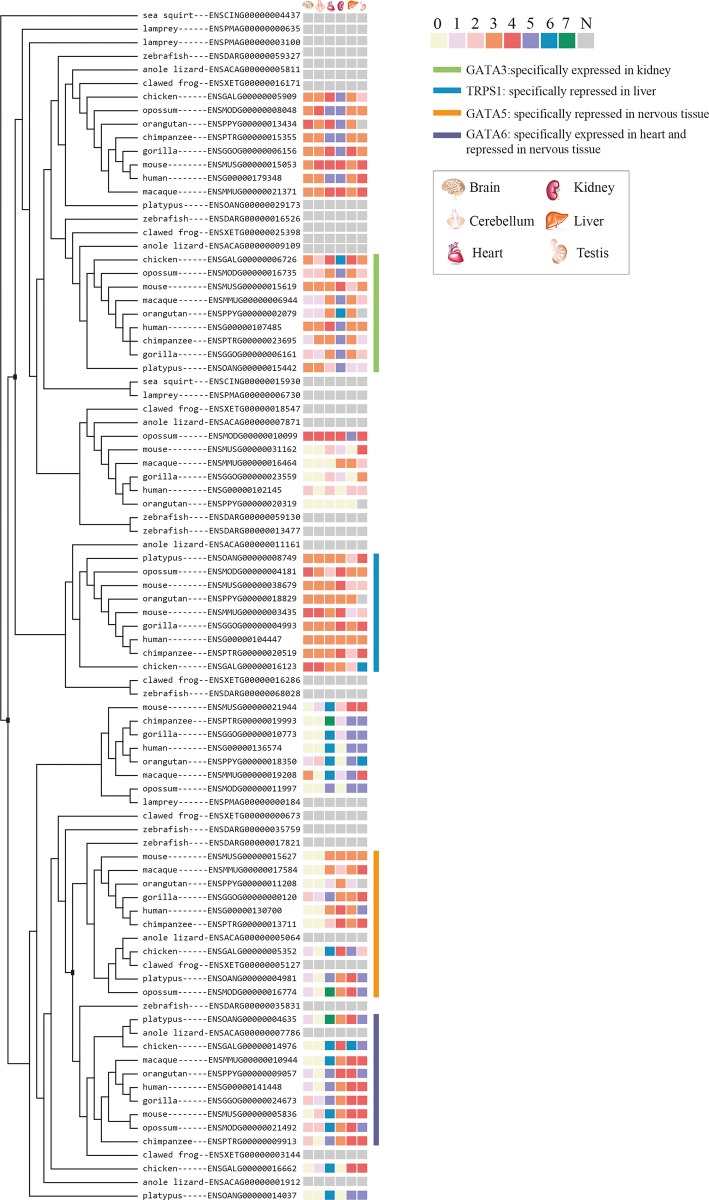
Phylogenetic tree of GATA transcription factor family. A *Ciona intestinalis* gene was selected as the outgroup to root the tree and only the cladogram is shown. The tree node where a possible large-scale duplication event happened is marked with a filled black square ■. 7 means the gene’s expression level is higher than 95% of all genes expressed in the organ. 6 is between 95% and 85%. 5 is between 85% and 65%. 4 is between 65% and 35%. 3 is between 35% and 15%. 2 is between 15% and 5%. 1 is lower than 5%. 0 means no expression at all. N is not available.

We discriminated the transcription factors from all OSER genes. These transcription factors demonstrate a clear organ/tissue-specific expression or repression pattern (Table 3). Some organ/tissue-specific transcription factors in different organs/tissues are from the same gene family, e.g. GATA transcription factor family (Fig. 5).

**Table 3:** Organ-specifically expressed or repressed transcriptional factors.

Organ/Tissue	Organ-specifically expressed TF	Organ-specifically repressed TF
Brain	BCL11A, TSHZ3, MKX, ZEB2, SATB1	HBP1, KLF11
Cerebellum	BAZ1B, FOXJ2, CTCF, FUBP1, ZFPM2, CHD7, ZNF385C	KLF10
Nervous tissue	CRTC1, ZNF365, TEF, CAMTA1, SOX2, MYT1L	ELF1, GATA5, GATA6, ZNF217, TGIF1
Heart	TBX18, GATA6, ZNF366	SALL1
Kidney	TFEC, GATA3, SIM1, TFCP2L1, EHF, ELF5, ELF3, FOXI1	ZFPM2
Liver	CREB3L3, TBX3, NRBF2	MYEF2, GLI3, SOX9, ZNF462, ZNF827, CAMTA1, ZNF536, TRPS1
Testis	TCFL5, TBX4, BAZ2B, E2F8, TMF1, LZTFL1, E2F7, ZNF217, HSF5, CNOT10, ZNF438, MTF1, BAZ1A, TRAF3IP1, NR6A1, TBPL1	TFEB, NFIB, CTBP1, KLF15, IRF2, PPARGC1A, JDP2

**Figure 6:**
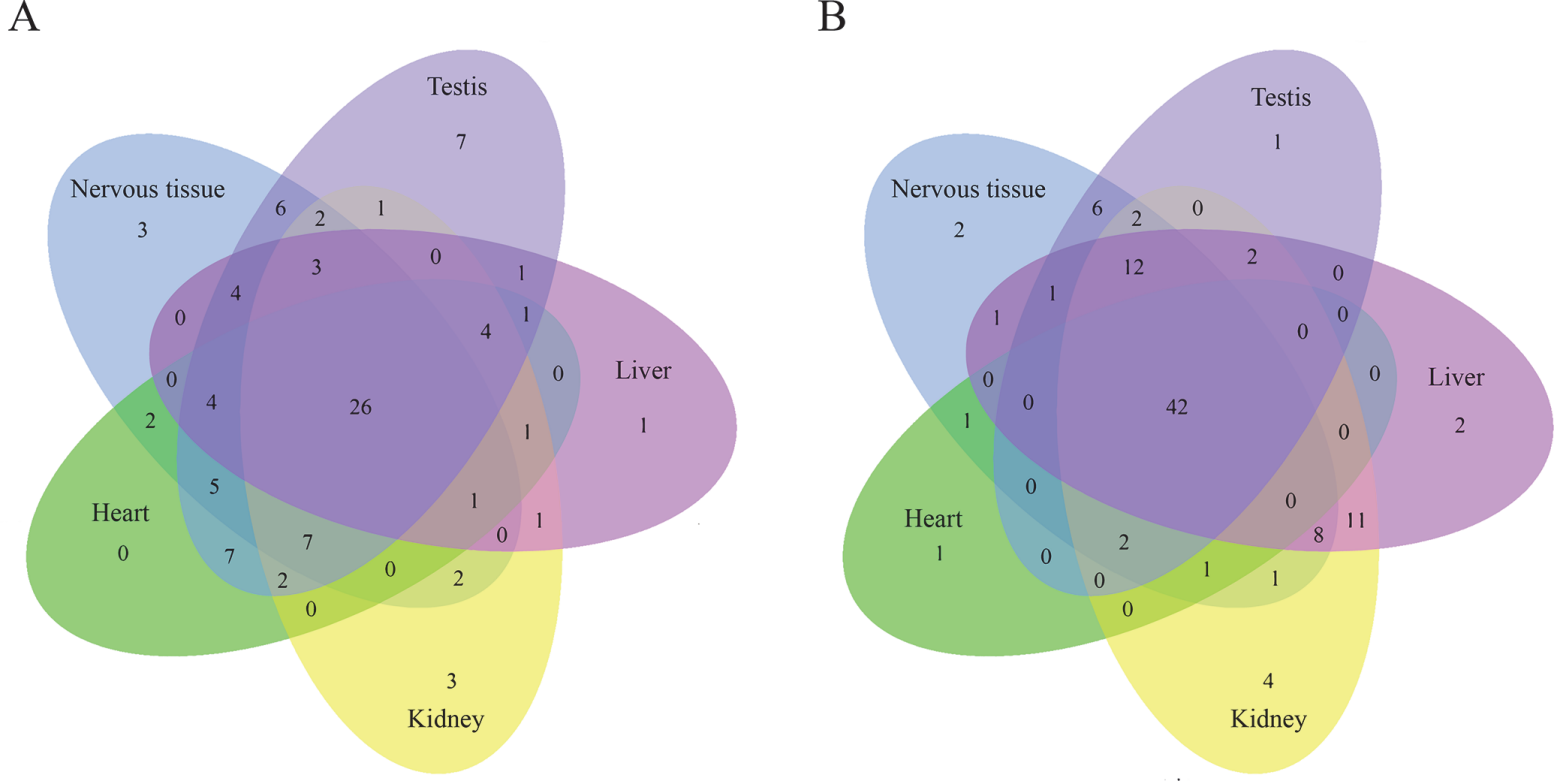
Venn diagram of transcription-related factors in five organs/tissues. A, the proximal-binding transcription-related factors in five organs/tissues. B, the distal-binding transcription-related factors in five organs/tissues. Nervous tissue includes the transcription-related factors in brain and cerebellum.

The ENCODE project provides us the information of the human transcriptional regulatory network [20]. We used its ChIP-seq data to find the transcription-related factors which might control the expression of OSER genes. Because the ENCODE data are restricted to the human genome, the transcription-related factors we found are based on human OSER genes. In 119 transcription-related factors from the ENCODE project, 114 of them might be involved in regulating human OSER genes. We listed five most common transcription-related factors for each specifically expressed gene group in Table 4. Some of them such as REST (RE1-silencing transcription factor) are organ/tissue-specific, but most of them show no organ/tissue specificity. 26 proximal-binding and 42 distal-binding transcription-related factors are shared by all organs/tissues in this study (Fig. 6A and 6B). It shows that the proximal-binding transcription factors are more diversified than the distal-binding ones across different organs/tissues. EP300, CTCF and RAD21 are the three most common transcription-related factors shared by all six organs. They have a relatively high expression level in all of them (S1, S2 and S3 Figs.).

**Table 4:** Five most common transcription-related factors might be involved in regulating OSER genes.

OSER gene group	Proximal binding TFs (the number of OSER genes it might control)	Distal binding TFs (the number of OSER genes it might control)
Brain-specifically expressed genes	REST(9), RAD21(7), CTCF(5), FOXA1(3), ZZZ3(3)	EP300(10), CTCF(3), SPI1(3), RAD21(2), TCF12 (2)
Cerebellum-specifically expressed genes	REST(5), CTCF(2), TAL1(2), E2F1(2), GATA2(2)	EP300(5), RAD21(2), CEBPB(2), SP1(2), STAT3(2)
Nervous-tissue-specifically expressed genes	REST(19), CTCF(15), RAD21(12), ZNF263(7), SUZ12(6)	EP300(30), SPI1(11), RAD21(10), SP1(10), CTCF(9)
Heart-specifically expressed genes	CTCF(8), GATA2(7), ESR1(7), TCF4(5), E2F1(4)	EP300(22), CTCF(6), CEBPB(5), RAD21(4), YY1(4)
Kidney-specifically expressed genes	RAD21(7), GATA2(5), CTCF(5), ESR1(5), TFAP2C(4)	EP300(24), SPI1(12), EBF1(11), RAD21(10), BATF(10)
Liver-specifically expressed genes	FOXA1(20), FOXA2(19), EP300(14), HNF4A(13), TCF4(13)	EP300 (33), TAL1(22), JUND(19), GATA2(18), POL2(17)
Testis-specifically expressed genes	E2F4(42), E2F1(22), CTCF(21), MYC(15), NFYA (14)	EP300(29), FOXA2(12), RAD21(12), FOXA1(12), JUND(11)

We compared the OSER gene binding sites of human CTCF and EP300 with mouse CTCF, mouse EP300 and chicken CTCF. The results are shown in Table 5. The CTCF binding overlap between human and mouse is around 50% in OSER genes. The EP300 binding overlap between human and mouse is less than 50% in OSER genes. The CTCF proximal binding overlap between human and chicken is less than 10% in OSER genes.

**Table 5:** Comparison of human ChIPseq data with mouse/chicken ChIPseq data.

Transcription factor	Number of proximal binding OSER gene	Proximal binding overlap with human	Number of distal binding OSER gene	Distal binding overlap with human
Human CTCF	72		51	
Mouse CTCF	704	49 (68%)	819	25 (49%)
Chicken CTCF	95	4 (5.6%)	291	15 (29%)
Human EP300	28		172	
Mouse EP300	291	9 (32%)	563	78 (45%)

## Discussion

We identified OSER clusters through multiple pairwise ANOVA comparisons. Due to the limitation of the statistical method employed in this study, we couldn’t guarantee that all genes in one OSER cluster have a perfectly uniform expression pattern. So we used functional enrichment analysis to validate their functions. In all specifically expressed genes, only cerebellum-specifically expressed ones didn’t produce a statistically meaningful DAVID result, although Benjamini-corrected FDR is a pretty stringent criterion. It is probably due to the small number of cerebellum-specific genes we used for DAVID analysis or the insufficient annotations of their functions in DAVID database. The function of cerebellum was once thought to be merely associated with motor function, but there is evidence that it may be also related to cognition and affection [23].

OSER genes don’t necessarily have a very high or very low expression level in their corresponding organ/tissue. For example, RE1-sliencing transcription factor (REST) represses the expression of neural genes in non-nervous tissues [24–26]. It shows a clearly repressed expression pattern in nervous tissues (S4 Fig.). In human brain and cerebellum, its expression level is lower than 65% of the genes expressed in these two organs. However, in human testis where REST has the highest expression quantile, its expression level is still lower than half of the genes expressed in human testis (data not shown). A lot of genes like REST may perform specific functions in one organ/tissue through a relatively low expression level. Because it is impossible to identify these genes by their expression levels in one organ/tissue, we focused on gene expression specificity instead of gene expression level in this study.

The expression profile of an organ expectedly involves the expression and repression of a variety of genes. Testis has the largest number of OSER genes in this study. The research has suggested that the wide expression spectrum of testis makes it a possible birthplace for new genes [27]. Liver is a little bit special from the other organs in terms of its expression profile. It has more repressed orthologous genes than expressed ones. While the functional enrichment analyses of most organ-specifically repressed gene groups yield no meaningful results, the DAVID result of liver-specifically repressed genes shows that their cellular components are mostly related to cytoskeleton, which provides the cell with structure and shape. It partly explains the molecular basis behind liver regeneration capability. Moreover, the DAVID result links their biological process to neuron projection development. It suggests that a portion of genes specifically expressed in nervous system are repressed in liver. Our analysis shows that 18 nervous-tissue-specifically expressed clusters are specifically repressed in liver, although it is not clear how these genes are transcriptionally regulated in both tissues (supporting information files).

After the establishment of major organ systems in the vertebrate common ancestor, vertebrate organs/tissues seemingly began to evolve in a gradual manner. Newly emerged genes were continuously integrated to the expression spectrum of each organ during the course of evolution. Our results propose that heart and kidney had stopped to integrate newly emerged genes into their expression spectra since the appearance of Amniota (lizard or chicken). Heart and kidney might have perfected their physiological functions before amniotic vertebrates entered the stage of evolution, which explains why heart and kidney stopped in integrating newly emerged genes earlier than the other organs in this study. Nervous tissues, liver and testis are still active in integrating newly emerged genes to their expression spectra after the appearance of Amniota, which suggests that these genes might be important for the evolution of amniotic vertebrates.

Our analyses show that once a gene occupied an expression niche in an organ, its evolutionary rate would be substantially shaped by the organ. Testis- and liver-specifically expressed genes averagely evolve more than twice as fast as the ones in nervous tissues. This phenomenon is also coined as tissue-driven genomic evolution [28]. The specifically expressed genes usually evolve faster than the repressed ones within one organ, but in nervous tissues the opposite is true. It proposes that nervous-tissue-specifically expressed genes are under strong purifying selection. The DAVID analysis of liver-specifically expressed genes shows that their biological processes are mostly involved in innate immune responses. The immune genes have to evolve fast to keep up with the rapid evolutionary speed of infectious agents such as viruses [29–31]. The selection pressure working on an individual organism is partaken by its thousands of genes. The physiological function of an organ is literally an evolutionary interface between natural selection and the genome. Different physiological function means different functional constraint. The selection pressure changes when it goes through different functional constraints. This change is finally reflected by the various evolutionary rates of the genes which support the physiological functions of different organs.

About 80% of OSER clusters (1210/1521) can be evolutionarily traced to sea squirt whose body system is underdeveloped compared to vertebrates. The studies of vertebrate genomes show that the common vertebrate ancestor experienced two rounds of genome duplication events, which indicates the important role of gene/genome duplication events in vertebrate organ/tissue divergence [1–3]. Our result shows that more than one third of the gene families (335/899) with a chordate or more ancient origin possess only one orthologous cluster, which proposes that they didn’t experience any large-scale gene duplication event. However, many duplicate genes are rapidly lost from the genome due to functional redundancy and the accumulation of detrimental mutations [32, 33]. Gene-loss event could make the duplication event within a gene family untraceable.

For those duplicate genes retained in the genome, their expression divergence is always a major concern for evolutionary biologists. Our result shows that the gene families containing the OSER clusters from two or more than two different organs/tissues usually experienced more duplication events than those only containing the OSER clusters from a single organ/tissue. Our analysis also demonstrates that if a gene family has an organ/tissue-specific expression pattern, it generally experienced fewer duplication events than those without a specific pattern. These results are consistent with the idea that gene duplication is an important source of expression diversity and functional novelty [34–36]. We find that the expression specificities in some gene families such as GATA binding protein family, kinesin family, and tripartite motif-containing protein family were coupled with large-scale duplication events (supporting information files). It explains the quick organ/tissue divergence in vertebrate linage after its split from Urochordata and Cephalochordata. We suspect that GATA transcription factor, transcriptional intermediary factor (TRIM) and kinesin play a pivotal role in chordate development and the duplication of these genes rapidly created developmental novelty in vertebrates.

The molecular mechanism underlying the expression divergence between/among duplicate genes is poorly understood. The paralogue genes (CTCFL and RAD21L1) of CTCF and RAD21 display a suppressed expression pattern in six organs while the paralogue gene (CREBBP) of EP300 maintains a high expression level in six organs like EP300 (S1, S2 and S3 Figs.). The ENCODE project only investigated a limited number of human transcription-related factors and their possible target genes, but failed to deliver the information of how these transcription-related factors regulate the expression of their target genes. The comparison of the binding sites of human CTCF and EP300 with mouse CTCF an EP300 shows a very limited binding conservation in OSER genes. The binding conservation between human CTCF and chicken CTCF is very low in OSER genes. These results propose that organ-specifically expressed or repressed orthologous genes are regulated in various combinatorial fashions in different species, although their expression features are well preserved among these species. Despite the enormous effort dedicated to understand the genome biology of various organisms, there is still no well-established knowledge of the cis-regulatory elements and their binding transcription factors for most known metazoan genes [37–39]. We believe that the future study of non-coding sequence evolution will give us some insights into the expression fate of duplicate genes and the transcriptional network behind vertebrate organ/tissue formation.

The study of vertebrate testicular transcriptomes suggests that a part of testis-expressed genes may not have immediate testis-related functions [27]. Our analysis shows that the genes specifically expressed in testis usually have a fast evolutionary rate. These results propose that some genes expressed in testis could escape from functional constraint and are more likely to acquire new functions than the genes expressed in other organs/tissues. Our phylogenetic analyses of some open reading frames show that they first expressed in testis and later got expressed in other organs/tissues in the process of evolution (S5, S6 and S7 Figs.). Although their functions are unknown, it is very possible that these ORFs first got the transcriptional activity in testis and later developed new functions for other organs/tissues. We speculate that testis may not only promote the birth of new genes [27], but also may help some of its specifically expressed genes to develop new functions for other organs/tissues.

Our study actually raises more questions than answers. So many genome projects like the ENCODE project only shed light on the unknown things that we didn’t use to know. We sincerely hope that our study will provide some research clues to the things about which we don’t have a clue.

## Supporting Information

S1 Fig Phylogenetic tree of E1A binding protein p300 family. A *Ciona intestinalis* gene was selected as the outgroup to root the tree and only the cladogram is shown. The tree node where a possible genome duplication event happened is marked with a filled black square ■. The numbers behind taxonomic unit (gene) are the gene’s expression ranks in different organs. The order of the numbers represents the order of organs as follows: brain, cerebellum, heart, kidney, liver and testis. 7 means the gene’s expression level is higher than 95% of all genes expressed in a specific organ within one species. 6 means the gene’s expression level is between 95% and 85% expression percentile in a specific organ within one species. 5 is between 85% and 65%. 4 is between 65% and 35%. 3 is between 35% and 15%. 2 is between 15% and 5%. 1 is lower than 5%. 0 means no expression at all. N is not available.
(TIF)

S2 Fig Phylogenetic tree of CCCTC-binding factor family. A *Ciona intestinalis* gene was selected as the outgroup to root the tree and only the cladogram is shown. The tree node where a possible genome duplication event happened is marked with a filled black square ■. The numbers behind taxonomic unit (gene) are the gene’s expression ranks in different organs. The order of the numbers represents the order of organs as follows: brain, cerebellum, heart, kidney, liver and testis. 7 means the gene’s expression level is higher than 95% of all genes expressed in a specific organ within one species. 6 means the gene’s expression level is between 95% and 85% expression percentile in a specific organ within one species. 5 is between 85% and 65%. 4 is between 65% and 35%. 3 is between 35% and 15%. 2 is between 15% and 5%. 1 is lower than 5%. 0 means no expression at all. N is not available.
(TIF)

S3 Fig Phylogenetic tree of rad21 homolog family. The *Ciona intestinalis* gene was used as the outgroup to root the tree and only the cladogram is shown. The tree node where a possible genome duplication event happened is marked with a filled black square ■. The numbers behind taxonomic unit (gene) are the gene’s expression ranks in different organs. The order of the numbers represents the order of organs as follows: brain, cerebellum, heart, kidney, liver and testis. 7 means the gene’s expression level is higher than 95% of all genes expressed in a specific organ within one species. 6 means the gene’s expression level is between 95% and 85% expression percentile in a specific organ within one species. 5 is between 85% and 65%. 4 is between 65% and 35%. 3 is between 35% and 15%. 2 is between 15% and 5%. 1 is lower than 5%. 0 means no expression at all. N is not available.
(TIF)

S4 Fig Phylogenetic tree of RE1-silencing transcription factor family. A *Ciona intestinalis* gene was selected as the outgroup to root the tree and only the cladogram is shown. The tree node where a possible genome duplication event happened is marked with a filled black square ■. The numbers behind taxonomic unit (gene) are the gene’s expression ranks in different organs. The order of the numbers represents the order of organs as follows: brain, cerebellum, heart, kidney, liver and testis. 7 means the gene’s expression level is higher than 95% of all genes expressed in a specific organ within one species. 6 means the gene’s expression level is between 95% and 85% expression percentile in a specific organ within one species. 5 is between 85% and 65%. 4 is between 65% and 35%. 3 is between 35% and 15%. 2 is between 15% and 5%. 1 is lower than 5%. 0 means no expression at all. N is not available.
(TIF)

S5 Fig Phylogenetic tree of chromosome 1 open reading frame 94 family. The anole lizard gene was used as the outgroup to root the tree and only the cladogram is shown. The numbers behind taxonomic unit (gene) are the gene’s expression ranks in different organs. The order of the numbers represents the order of organs as follows: brain, cerebellum, heart, kidney, liver and testis. 7 means the gene’s expression level is higher than 95% of all genes expressed in a specific organ within one species. 6 means the gene’s expression level is between 95% and 85% expression percentile in a specific organ within one species. 5 is between 85% and 65%. 4 is between 65% and 35%. 3 is between 35% and 15%. 2 is between 15% and 5%. 1 is lower than 5%. 0 means no expression at all. N is not available.
(TIF)

S6 Fig Phylogenetic tree of chromosome 4 open reading frame 47 family. The *Ciona intestinalis* gene was used as the outgroup to root the tree and only the cladogram is shown. The numbers behind taxonomic unit (gene) are the gene’s expression ranks in different organs. The order of the numbers represents the order of organs as follows: brain, cerebellum, heart, kidney, liver and testis. 7 means the gene’s expression level is higher than 95% of all genes expressed in a specific organ within one species. 6 means the gene’s expression level is between 95% and 85% expression percentile in a specific organ within one species. 5 is between 85% and 65%. 4 is between 65% and 35%. 3 is between 35% and 15%. 2 is between 15% and 5%. 1 is lower than 5%. 0 means no expression at all. N is not available.
(TIF)

S7 Fig Phylogenetic tree of chromosome 9 open reading frame 96 family. The *Ciona intestinalis* gene was used as the outgroup to root the tree and only the cladogram is shown. The numbers behind taxonomic unit (gene) are the gene’s expression ranks in different organs. The order of the numbers represents the order of organs as follows: brain, cerebellum, heart, kidney, liver and testis. 7 means the gene’s expression level is higher than 95% of all genes expressed in a specific organ within one species. 6 means the gene’s expression level is between 95% and 85% expression percentile in a specific organ within one species. 5 is between 85% and 65%. 4 is between 65% and 35%. 3 is between 35% and 15%. 2 is between 15% and 5%. 1 is lower than 5%. 0 means no expression at all. N is not available.
(TIF)

S1 Table DAVID functional annotation analysis of brain-specifically expressed genes. (DOC)

S2 Table DAVID functional annotation analysis of cerebellum-specifically expressed genes. (DOC)

S3 Table DAVID functional annotation analysis of heart-specifically expressed genes. (DOC)

S4 Table DAVID functional annotation analysis of kidney-specifically expressed genes. (DOC)

S5 Table DAVID functional annotation analysis of liver-specifically expressed genes. (DOC)

S6 Table DAVID functional annotation analysis of liver specifically-repressed genes. (DOC)

S7 Table DAVID functional annotation analysis of nervous-tissue-specifically expressed genes. (DOC)

S8 Table DAVID functional annotation analysis of testis-specifically expressed genes. (DOC)

S9 Table DAVID functional annotation analysis of testis specifically-repressed genes. (DOC)
