# Supplementary figures and images for: The Evolutionary Panorama of Organ-Specifically Expressed or Repressed Orthologous Genes in Nine Vertebrate Species
Source: PLoS One. 2015 Feb 13;10(2):e0116872. doi: 10.1371/journal.pone.0116872 (PMC4332667; doi:10.1371/journal.pone.0116872)

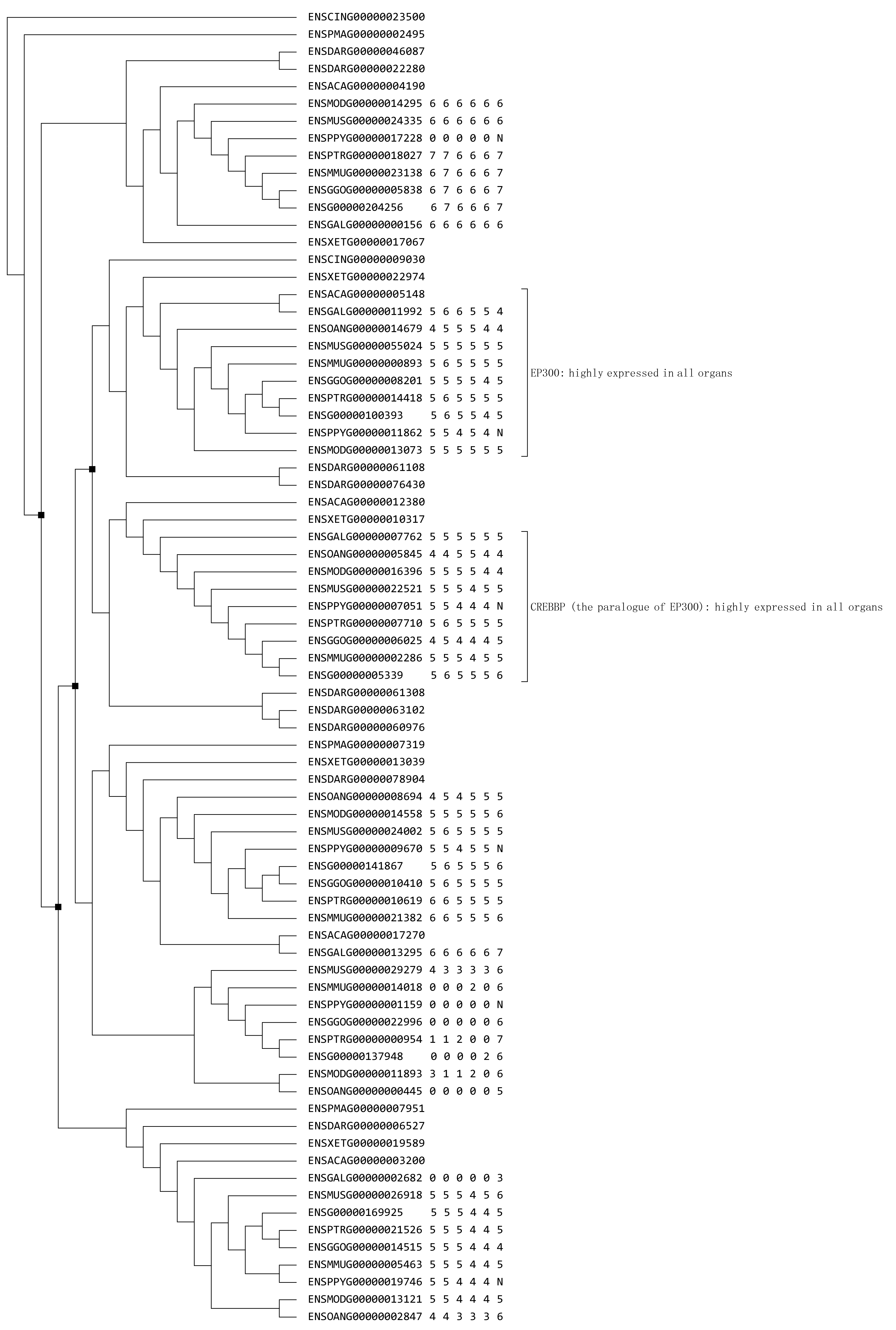

Supplement: S1 Fig — A Ciona intestinalis gene was selected as the outgroup to root the tree and only the cladogram is shown. The tree node where a possible genome duplication event happened is marked with a filled black square ■. The numbers behind taxonomic unit (gene) are the gene’s expression ranks in different organs. The order of the numbers represents the order of organs as follows: brain, cerebellum, heart, kidney, liver and testis. 7 means the gene’s expression level is higher than 95% of all genes expressed in a specific organ within one species. 6 means the gene’s expression level is between 95% and 85% expression percentile in a specific organ within one species. 5 is between 85% and 65%. 4 is between 65% and 35%. 3 is between 35% and 15%. 2 is between 15% and 5%. 1 is lower than 5%. 0 means no expression at all. N is not available. (TIF) [file pone.0116872.s001.tif]

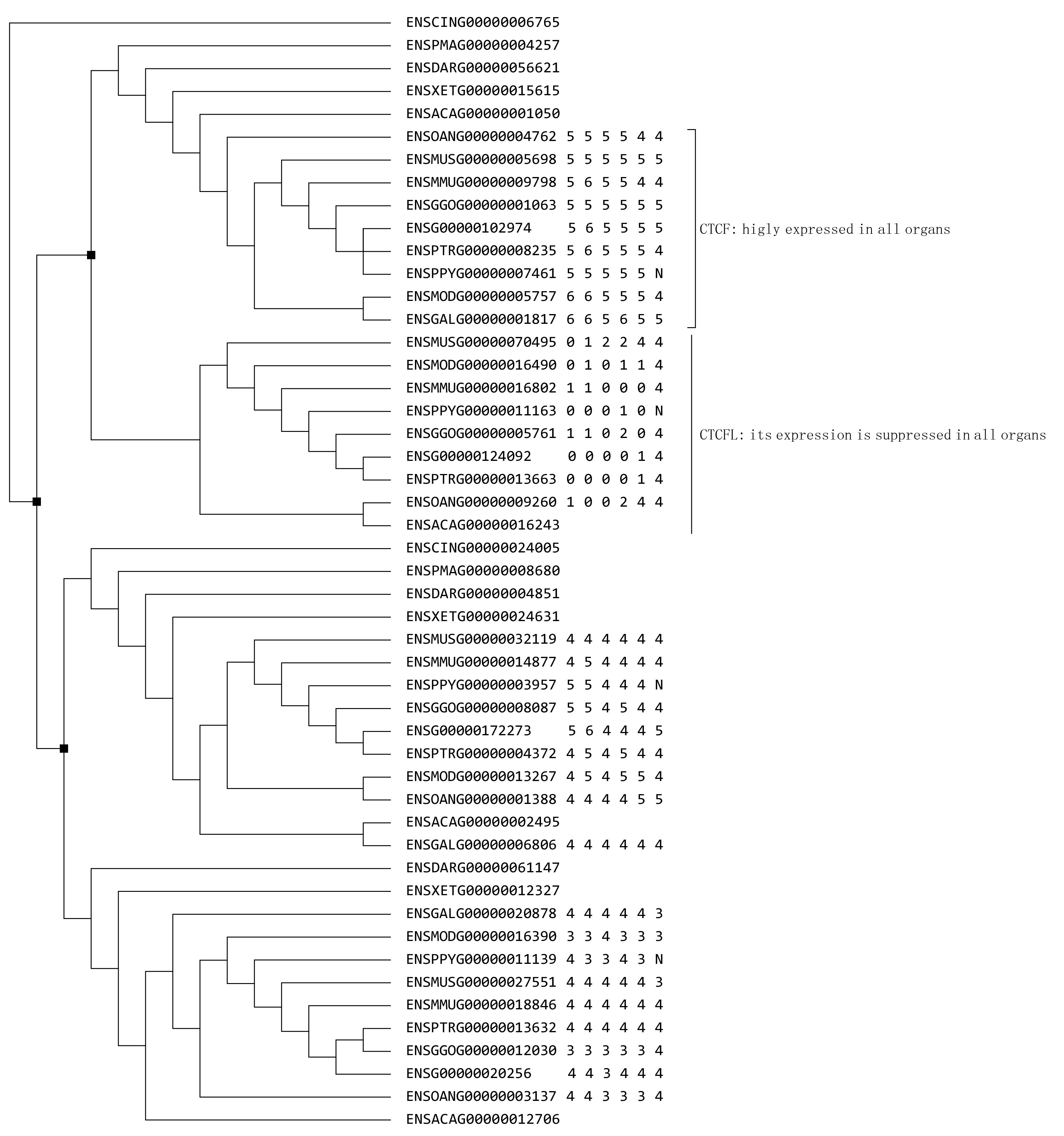

Supplement: S2 Fig — A Ciona intestinalis gene was selected as the outgroup to root the tree and only the cladogram is shown. The tree node where a possible genome duplication event happened is marked with a filled black square ■. The numbers behind taxonomic unit (gene) are the gene’s expression ranks in different organs. The order of the numbers represents the order of organs as follows: brain, cerebellum, heart, kidney, liver and testis. 7 means the gene’s expression level is higher than 95% of all genes expressed in a specific organ within one species. 6 means the gene’s expression level is between 95% and 85% expression percentile in a specific organ within one species. 5 is between 85% and 65%. 4 is between 65% and 35%. 3 is between 35% and 15%. 2 is between 15% and 5%. 1 is lower than 5%. 0 means no expression at all. N is not available. (TIF) [file pone.0116872.s002.tif]

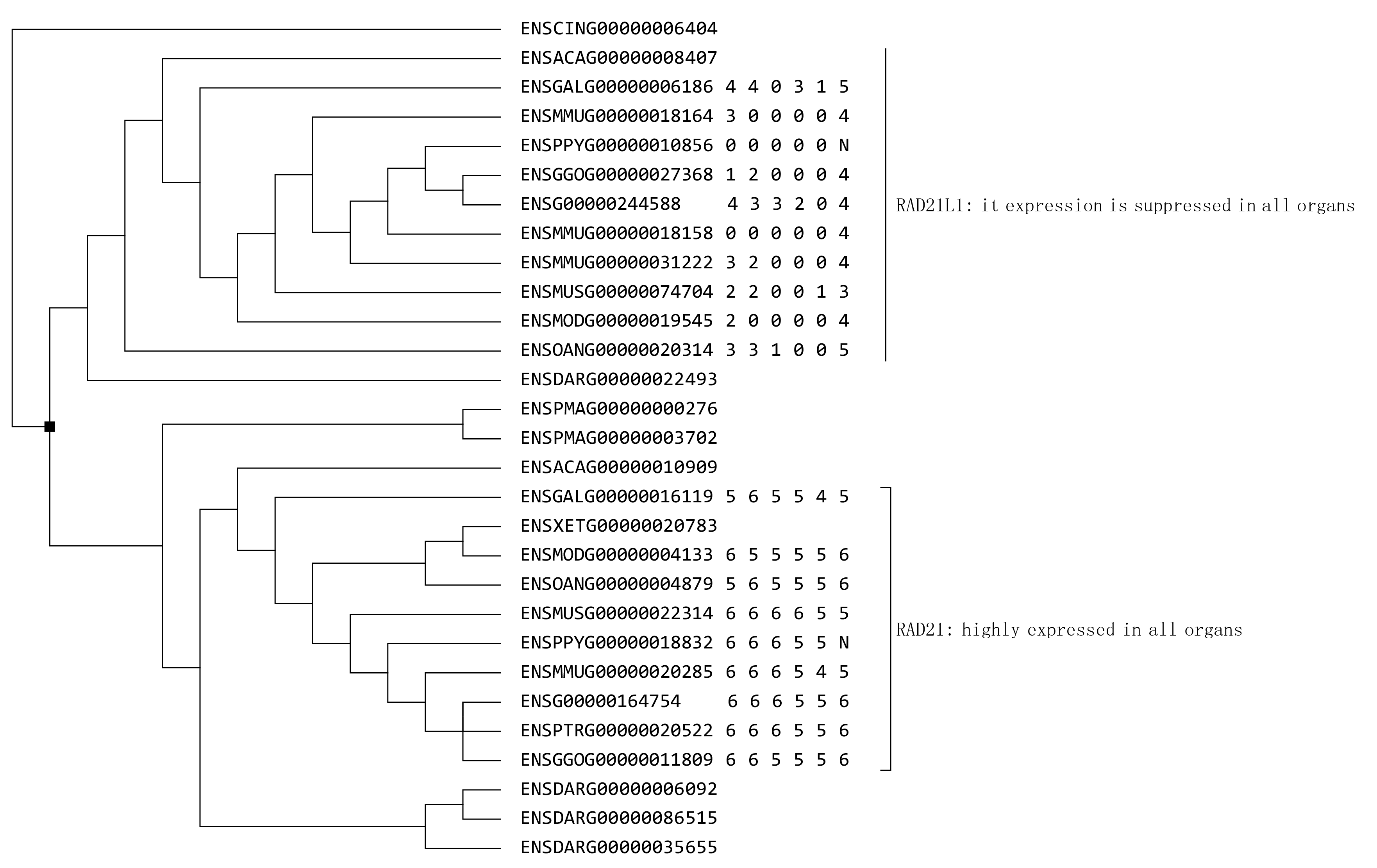

Supplement: S3 Fig — The Ciona intestinalis gene was used as the outgroup to root the tree and only the cladogram is shown. The tree node where a possible genome duplication event happened is marked with a filled black square ■. The numbers behind taxonomic unit (gene) are the gene’s expression ranks in different organs. The order of the numbers represents the order of organs as follows: brain, cerebellum, heart, kidney, liver and testis. 7 means the gene’s expression level is higher than 95% of all genes expressed in a specific organ within one species. 6 means the gene’s expression level is between 95% and 85% expression percentile in a specific organ within one species. 5 is between 85% and 65%. 4 is between 65% and 35%. 3 is between 35% and 15%. 2 is between 15% and 5%. 1 is lower than 5%. 0 means no expression at all. N is not available. (TIF) [file pone.0116872.s003.tif]

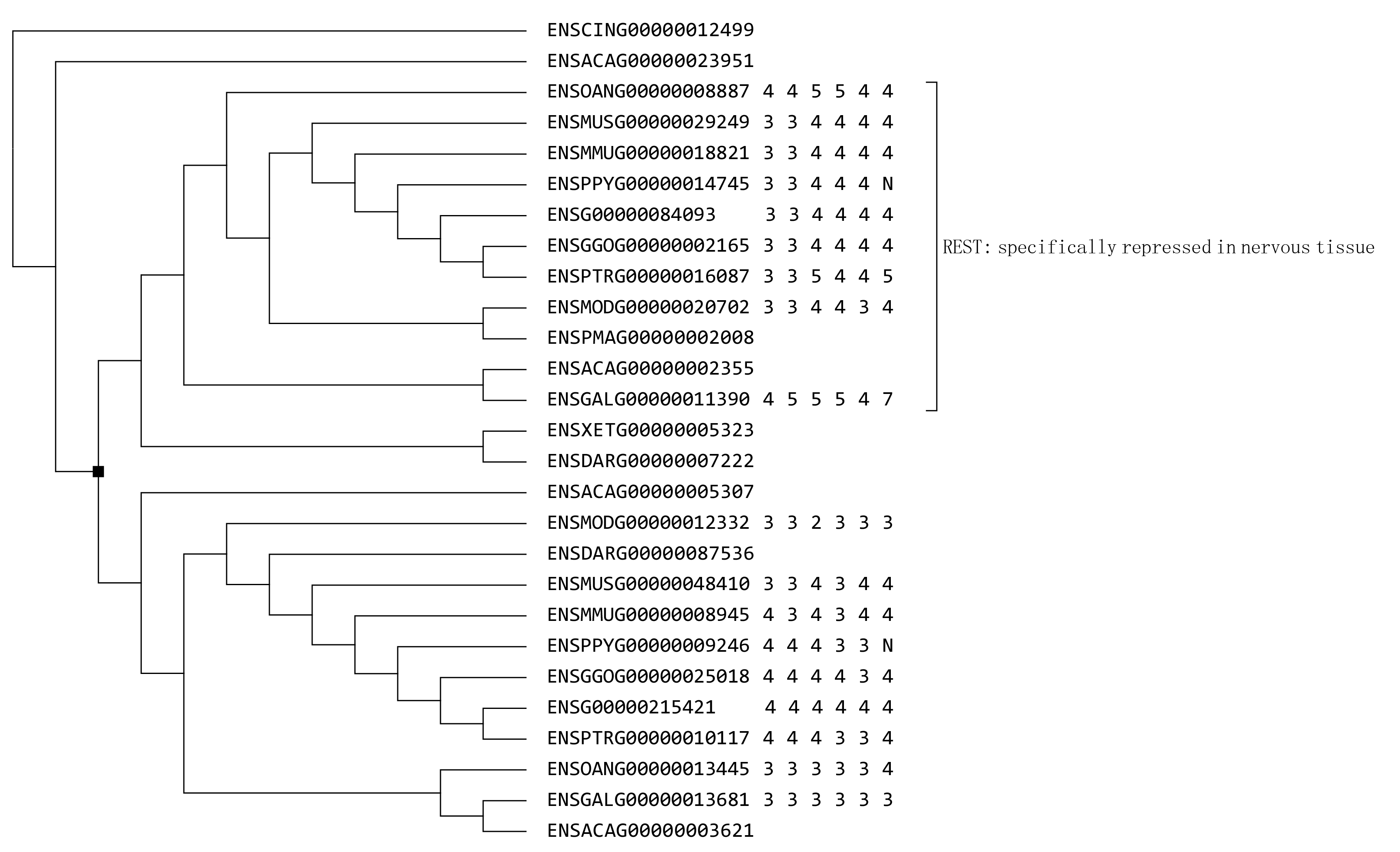

Supplement: S4 Fig — A Ciona intestinalis gene was selected as the outgroup to root the tree and only the cladogram is shown. The tree node where a possible genome duplication event happened is marked with a filled black square ■. The numbers behind taxonomic unit (gene) are the gene’s expression ranks in different organs. The order of the numbers represents the order of organs as follows: brain, cerebellum, heart, kidney, liver and testis. 7 means the gene’s expression level is higher than 95% of all genes expressed in a specific organ within one species. 6 means the gene’s expression level is between 95% and 85% expression percentile in a specific organ within one species. 5 is between 85% and 65%. 4 is between 65% and 35%. 3 is between 35% and 15%. 2 is between 15% and 5%. 1 is lower than 5%. 0 means no expression at all. N is not available. (TIF) [file pone.0116872.s004.tif]

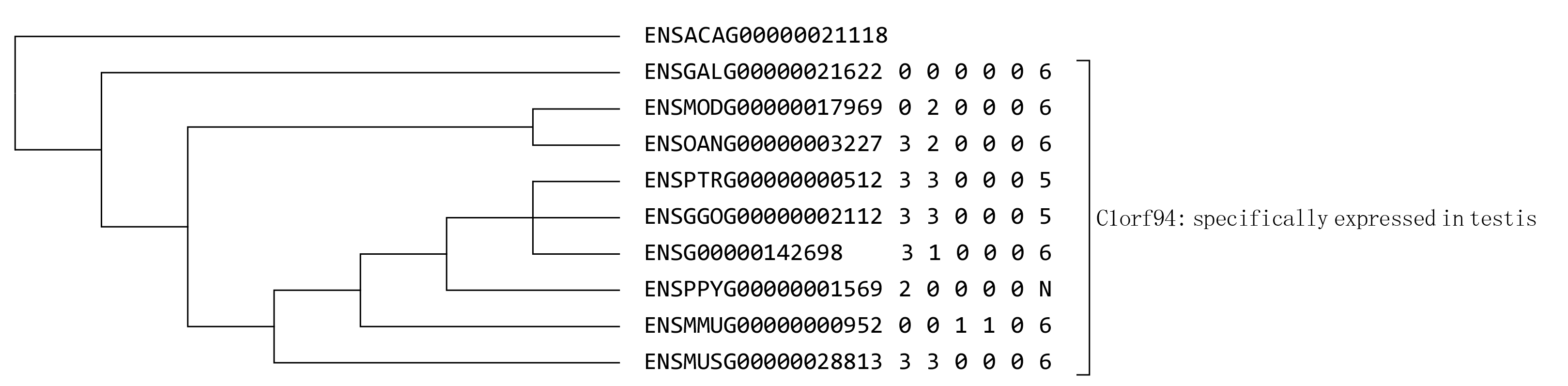

Supplement: S5 Fig — The anole lizard gene was used as the outgroup to root the tree and only the cladogram is shown. The numbers behind taxonomic unit (gene) are the gene’s expression ranks in different organs. The order of the numbers represents the order of organs as follows: brain, cerebellum, heart, kidney, liver and testis. 7 means the gene’s expression level is higher than 95% of all genes expressed in a specific organ within one species. 6 means the gene’s expression level is between 95% and 85% expression percentile in a specific organ within one species. 5 is between 85% and 65%. 4 is between 65% and 35%. 3 is between 35% and 15%. 2 is between 15% and 5%. 1 is lower than 5%. 0 means no expression at all. N is not available. (TIF) [file pone.0116872.s005.tif]

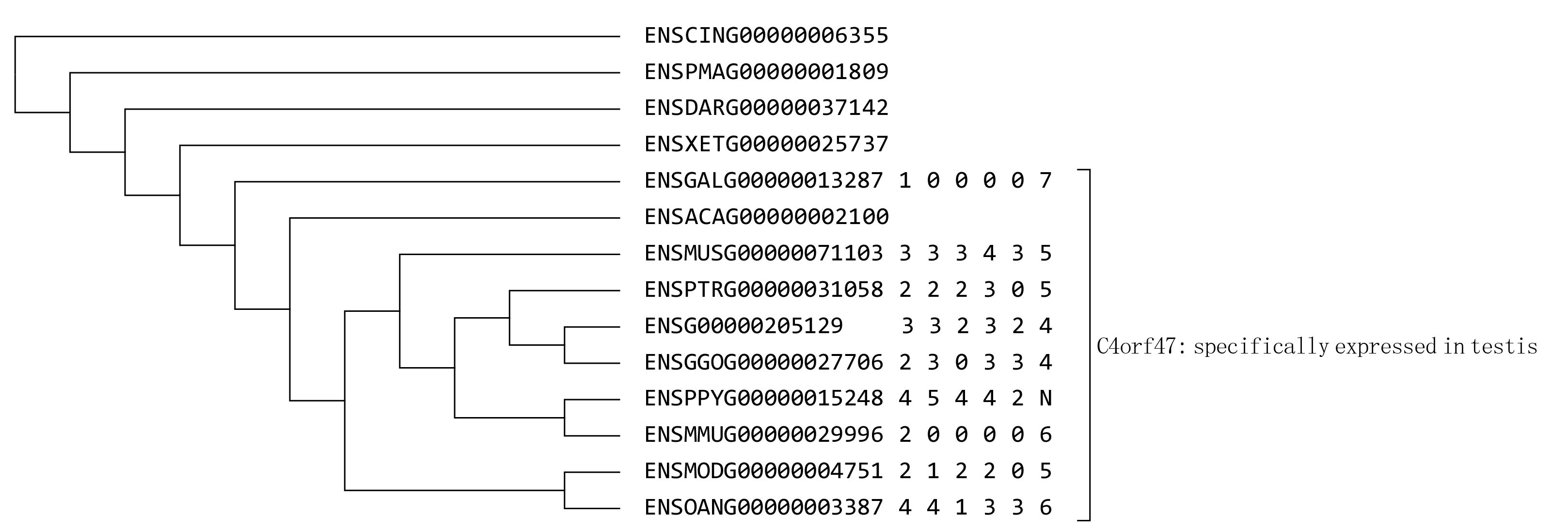

Supplement: S6 Fig — The Ciona intestinalis gene was used as the outgroup to root the tree and only the cladogram is shown. The numbers behind taxonomic unit (gene) are the gene’s expression ranks in different organs. The order of the numbers represents the order of organs as follows: brain, cerebellum, heart, kidney, liver and testis. 7 means the gene’s expression level is higher than 95% of all genes expressed in a specific organ within one species. 6 means the gene’s expression level is between 95% and 85% expression percentile in a specific organ within one species. 5 is between 85% and 65%. 4 is between 65% and 35%. 3 is between 35% and 15%. 2 is between 15% and 5%. 1 is lower than 5%. 0 means no expression at all. N is not available. (TIF) [file pone.0116872.s006.tif]

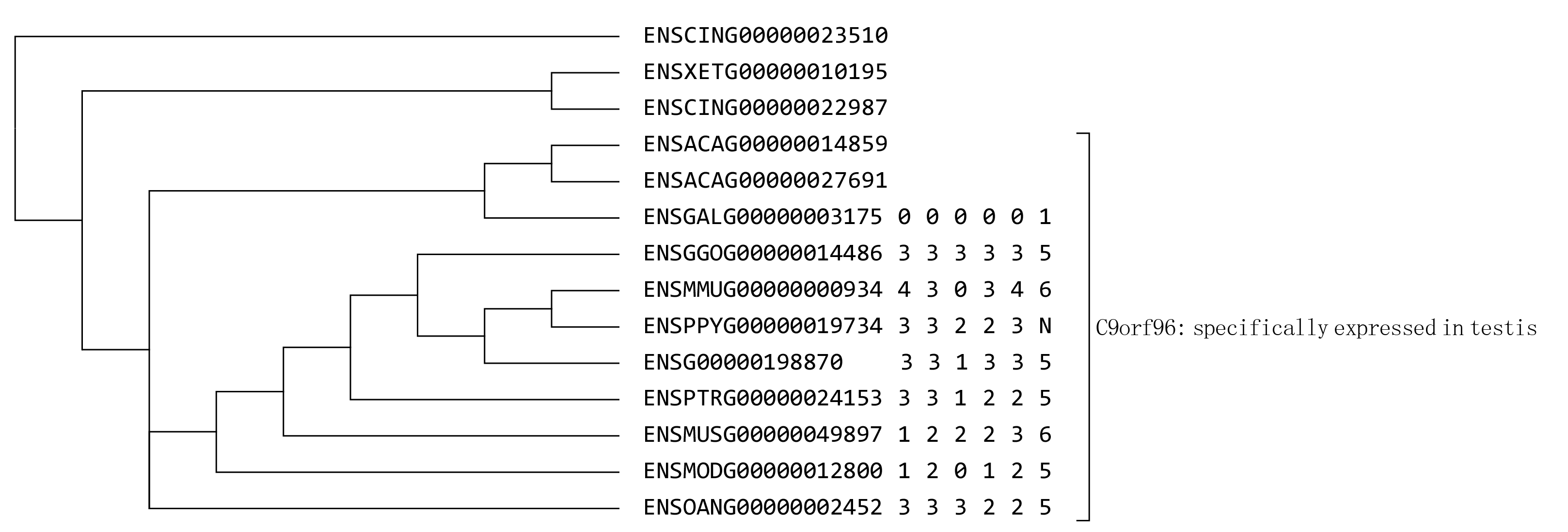

Supplement: S7 Fig — The Ciona intestinalis gene was used as the outgroup to root the tree and only the cladogram is shown. The numbers behind taxonomic unit (gene) are the gene’s expression ranks in different organs. The order of the numbers represents the order of organs as follows: brain, cerebellum, heart, kidney, liver and testis. 7 means the gene’s expression level is higher than 95% of all genes expressed in a specific organ within one species. 6 means the gene’s expression level is between 95% and 85% expression percentile in a specific organ within one species. 5 is between 85% and 65%. 4 is between 65% and 35%. 3 is between 35% and 15%. 2 is between 15% and 5%. 1 is lower than 5%. 0 means no expression at all. N is not available. (TIF) [file pone.0116872.s007.tif]
