# Supplementary material for: The Evolutionary Panorama of Organ-Specifically Expressed or Repressed Orthologous Genes in Nine Vertebrate Species
Source: PLoS One. 2015 Feb 13;10(2):e0116872. doi: 10.1371/journal.pone.0116872 (PMC4332667; doi:10.1371/journal.pone.0116872)
Supplement: S1 Table — (DOC) [file pone.0116872.s008.doc]

**Table S1.** DAVID functional annotation analysis of brain-specifically expressed genes.

| Category | Term | Benjamini-corrected FDR |
| --- | --- | --- |
| Go: Biological process | neuron development | 1.4E-6 |
|  | neuron differentiation | 5.2E-6 |
|  | neuron projection development | 5.2E-6 |
|  | axonogenesis | 1.1E-5 |
|  | cell morphogenesis involved in neuron differentiation | 2.1E-5 |
|  | neuron projection morphogenesis | 2.1E-5 |
|  | cell morphogenesis involved in differentiation | 7.9E-5 |
|  | cell projection morphogenesis | 7.3E-5 |
|  | synaptic transmission | 7.9E-5 |
| Go: Cellular component | neuron projection | 6.3E-12 |
|  | plasma membrane | 1.6E-9 |
|  | dendrite | 3.7E-8 |
|  | cell projection | 5.5E-8 |
|  | plasma membrane part | 2.4E-7 |
|  | axon | 1.8E-6 |
|  | synapse part | 2.2E-6 |
| Go: Molecular function | amine receptor activity | 2.1E-4 |
|  | gated channel activity | 9.2E-4 |
|  | ion channel activity | 1.1E-3 |
|  | voltage-gated potassium channel activity | 9.9E-4 |
|  | substrate specific channel activity | 9.3E-4 |
|  | voltage-gated cation channel activity | 1.0E-3 |
| KEGG pathway | Neuroactive ligand-receptor interaction | 4.1E-7 |
|  | Calcium signaling pathway | 2.8E-5 |
