# Supplementary material for: The Evolutionary Panorama of Organ-Specifically Expressed or Repressed Orthologous Genes in Nine Vertebrate Species
Source: PLoS One. 2015 Feb 13;10(2):e0116872. doi: 10.1371/journal.pone.0116872 (PMC4332667; doi:10.1371/journal.pone.0116872)
Supplement: S2 Table — (DOC) [file pone.0116872.s009.doc]

**Table S2.** DAVID functional annotation analysis of cerebellum-specifically expressed genes.

| Category | Term | Benjamini-corrected FDR |
| --- | --- | --- |
| Go: Biological process | negative regulation of gene expression | 9.7E-1* |
|  | negative regulation of macromolecule biosynthetic process | 9.2E-1* |
|  | negative regulation of cellular biosynthetic process | 8.5E-1* |
| Go: Cellular component | synapse | 2.2E-1* |
|  | synapse part | 1.2E-1* |
|  | postsynaptic density | 8.8E-2* |
|  | dendritic spine | 2.2E-1* |
| Go: Molecular function | transcription repressor activity | 9.3E-1* |
|  | metal ion binding | 8.5E-1* |
|  | cation binding | 8.0E-1* |
| KEGG pathway | Long-term depression | 3.7E-1* |

* Benjamini-corrected FDR is not statistically significant.
