# Supplementary material for: The Evolutionary Panorama of Organ-Specifically Expressed or Repressed Orthologous Genes in Nine Vertebrate Species
Source: PLoS One. 2015 Feb 13;10(2):e0116872. doi: 10.1371/journal.pone.0116872 (PMC4332667; doi:10.1371/journal.pone.0116872)
Supplement: S3 Table — (DOC) [file pone.0116872.s010.doc]

**Table S3.** DAVID functional annotation analysis of heart-specifically expressed genes.

| Category | Term | Benjamini-corrected FDR |
| --- | --- | --- |
| Go: Biological process | muscle system process | 3.7E-11 |
|  | muscle contraction | 6.6E-11 |
|  | muscle organ development | 7.4E-7 |
|  | heart development | 7.1E-7 |
|  | cytoskeleton organization | 2.2E-5 |
|  | response to hypoxia | 2.9E-4 |
|  | response to oxygen levels | 3.9E-4 |
|  | regulation of heart contraction | 4.5E-4 |
|  | striated muscle tissue development | 7.6E-4 |
| Go: Cellular component | contractile fiber | 4.5E-20 |
|  | myofibril | 1.1E-19 |
|  | contractile fiber part | 3.3E-18 |
|  | sarcomere | 6.0E-15 |
|  | I band | 7.8E-13 |
|  | actin cytoskeleton | 2.3E-11 |
|  | Z disc | 9.7E-11 |
|  | cytoskeleton | 3.8E-9 |
| Go: Molecular function | cytoskeletal protein binding | 7.2E-14 |
|  | actin binding | 1.1E-11 |
|  | structural constituent of muscle | 7.6E-6 |
|  | structural molecule activity | 7.8E-4 |
|  | actinin binding | 4.9E-3 |
| KEGG pathway | Arrhythmogenic right ventricular cardiomyopathy (ARVC) | 2.7E-4 |
|  | Hypertrophic cardiomyopathy (HCM) | 2.9E-4 |
|  | Dilated cardiomyopathy | 3.2E-3 |
|  | Cardiac muscle contraction | 9.5E-3 |
