# Supplementary material for: The Evolutionary Panorama of Organ-Specifically Expressed or Repressed Orthologous Genes in Nine Vertebrate Species
Source: PLoS One. 2015 Feb 13;10(2):e0116872. doi: 10.1371/journal.pone.0116872 (PMC4332667; doi:10.1371/journal.pone.0116872)
Supplement: S4 Table — (DOC) [file pone.0116872.s011.doc]

**Table S4.** DAVID functional annotation analysis of kidney-specifically expressed genes.

| Category | Term | Benjamini-corrected FDR |
| --- | --- | --- |
| Go: Biological process | sodium ion transport | 7.3E-10 |
|  | monovalent inorganic cation transport | 1.6E-8 |
|  | cation transport | 1.3E-8 |
|  | transmembrane transport | 1.9E-8 |
|  | ion transport | 2.7E-8 |
|  | metal ion transport | 9.7E-7 |
|  | secretion | 4.1E-6 |
|  | excretion | 2.8E-5 |
|  | response to nutrient levels | 7.3E-3 |
| Go: Cellular component | apical plasma membrane | 6.1E-17 |
|  | plasma membrane | 2.7E-11 |
|  | plasma membrane part | 4.0E-10 |
|  | intrinsic to plasma membrane | 2.0E-4 |
|  | integral to plasma membrane | 2.9E-4 |
|  | brush border | 2.7E-4 |
|  | integral to membrane | 2.4E-4 |
|  | intrinsic to membrane | 2.2E-4 |
|  | basolateral plasma membrane | 2.5E-4 |
| Go: Molecular function | sodium ion binding | 1.4E-9 |
|  | alkali metal ion binding | 1.5E-7 |
|  | symporter activity | 5.9E-6 |
|  | amine transmembrane transporter activity | 1.8E-2 |
|  | anion transmembrane transporter activity | 2.0E-2 |
|  | amino acid transmembrane transporter activity | 3.3E-2 |
| KEGG pathway | Aldosterone-regulated sodium reabsorption | 1.1E-5 |
