# Supplementary material for: The Evolutionary Panorama of Organ-Specifically Expressed or Repressed Orthologous Genes in Nine Vertebrate Species
Source: PLoS One. 2015 Feb 13;10(2):e0116872. doi: 10.1371/journal.pone.0116872 (PMC4332667; doi:10.1371/journal.pone.0116872)
Supplement: S5 Table — (DOC) [file pone.0116872.s012.doc]

**Table S5.** DAVID functional annotation analysis of liver-specifically expressed genes.

| Category | Term | Benjamini-corrected FDR |
| --- | --- | --- |
| Go: Biological process | response to wounding | 1.4E-22 |
|  | acute inflammatory response | 3.5E-19 |
|  | complement activation | 8.7E-18 |
|  | protein maturation by peptide bond cleavage | 6.8E-18 |
|  | activation of plasma proteins involved in acute inflammatory response | 8.2E-18 |
|  | protein processing | 5.9E-17 |
|  | protein maturation | 3.2E-16 |
|  | complement activation, classical pathway | 1.0E-15 |
|  | humoral immune response mediated by circulating immunoglobulin | 2.8E-15 |
|  | humoral immune response | 1.8E-14 |
|  | inflammatory response | 3.4E-14 |
|  | steroid metabolic process | 4.6E-14 |
| Go: Cellular component | extracellular space | 1.2E-24 |
|  | extracellular region | 1.5E-19 |
|  | extracellular region part | 2.3E-18 |
|  | vesicle lumen | 1.7E-11 |
|  | platelet alpha granule lumen | 9.5E-11 |
|  | cytoplasmic membrane-bounded vesicle lumen | 1.9E-10 |
|  | platelet alpha granule | 2.7E-9 |
|  | microsome | 2.2E-7 |
| Go: Molecular function | iron ion binding | 8.9E-8 |
|  | endopeptidase inhibitor activity | 6.7E-8 |
|  | peptidase inhibitor activity | 9.6E-8 |
|  | serine-type endopeptidase inhibitor activity | 1.5E-7 |
|  | lipid binding | 1.2E-7 |
|  | polysaccharide binding | 4.9E-7 |
|  | pattern binding | 4.9E-7 |
|  | glycosaminoglycan binding | 1.2E-6 |
| KEGG pathway | Complement and coagulation cascades | 1.9E-22 |
|  | Primary bile acid biosynthesis | 2.1E-5 |
