# Supplementary material for: The Evolutionary Panorama of Organ-Specifically Expressed or Repressed Orthologous Genes in Nine Vertebrate Species
Source: PLoS One. 2015 Feb 13;10(2):e0116872. doi: 10.1371/journal.pone.0116872 (PMC4332667; doi:10.1371/journal.pone.0116872)
Supplement: S6 Table — (DOC) [file pone.0116872.s013.doc]

**Table S6.** DAVID functional annotation analysis of liver specifically-repressed genes.

| Category | Term | Benjamini-corrected FDR |
| --- | --- | --- |
| Go: Biological process | cell projection organization | 1.6E-3 |
|  | cell morphogenesis | 9.4E-3 |
|  | cell projection morphogenesis | 7.3E-3 |
|  | cell part morphogenesis | 8.7E-3 |
|  | cellular component morphogenesis | 1.4E-2 |
|  | neuron projection development | 2.5E-2 |
|  | axonogenesis | 3.6E-2 |
|  | cell morphogenesis involved in differentiation | 5.0E-2 |
| Go: Cellular component | cytoskeleton | 5.0E-4 |
|  | neuron projection | 3.1E-3 |
|  | cell projection | 3.6E-2 |
| Go: Molecular function | calcium ion binding | 7.4E-2* |
|  | GTPase activator activity | 8.3E-2* |
|  | cytoskeletal protein binding | 7.4E-2* |
|  | lipoprotein binding | 8.1E-2* |
| KEGG pathway | Calcium signaling pathway | 3.4E-1* |
|  | Phosphatidylinositol signaling system | 4.1E-1* |

* Benjamini-corrected FDR is not statistically significant.
