# Supplementary material for: The Evolutionary Panorama of Organ-Specifically Expressed or Repressed Orthologous Genes in Nine Vertebrate Species
Source: PLoS One. 2015 Feb 13;10(2):e0116872. doi: 10.1371/journal.pone.0116872 (PMC4332667; doi:10.1371/journal.pone.0116872)
Supplement: S7 Table — (DOC) [file pone.0116872.s014.doc]

**Table S7.** DAVID functional annotation analysis of nervous-tissue-specifically expressed genes.

| Category | Term | Benjamini-corrected FDR |
| --- | --- | --- |
| Go: Biological process | synaptic transmission | 1.0E-15 |
|  | transmission of nerve impulse | 8.1E-14 |
|  | regulation of synaptic transmission | 8.6E-11 |
|  | cell-cell signaling | 1.0E-10 |
|  | ion transport | 2.0E-10 |
|  | regulation of transmission of nerve impulse | 2.2E-10 |
|  | regulation of neurological system process | 4.4E-10 |
|  | neuron differentiation | 1.1E-9 |
|  | neuron development | 1.1E-8 |
|  | cell morphogenesis involved in neuron differentiation | 2.5E-8 |
|  | neuron projection development | 4.8E-8 |
|  | axonogenesis | 1.7E-7 |
|  | neurotransmitter transport | 3.1E-7 |
|  | cell morphogenesis involved in differentiation | 3.9E-7 |
|  | neuron projection morphogenesis | 8.0E-7 |
|  | regulation of synaptic plasticity | 1.0E-6 |
| Go: Cellular component | synapse | 6.2E-19 |
|  | neuron projection | 3.1E-18 |
|  | synapse part | 3.1E-14 |
|  | cell junction | 5.9E-11 |
|  | plasma membrane | 6.2E-11 |
|  | axon | 3.1E-9 |
|  | cell projection | 5.2E-9 |
|  | plasma membrane part | 6.5E-8 |
|  | synaptic vesicle | 1.5E-7 |
| Go: Molecular function | substrate specific channel activity | 1.5E-10 |
|  | ion channel activity | 1.6E-10 |
|  | channel activity | 1.4E-10 |
|  | passive transmembrane transporter activity | 1.1E-10 |
|  | gated channel activity | 1.5E-10 |
|  | ligand-gated ion channel activity | 7.1E-8 |
|  | ligand-gated channel activity | 7.1E-8 |
|  | extracellular ligand-gated ion channel activity | 1.5E-6 |
|  | ionotropic glutamate receptor activity | 2.2E-6 |
|  | extracellular-glutamate-gated ion channel activity | 3.0E-6 |
|  | metal ion transmembrane transporter activity | 3.5E-6 |
|  | glutamate receptor activity | 6.4E-6 |
| KEGG pathway | Neuroactive ligand-receptor interaction | 9.3E-4 |
|  | Axon guidance | 1.1E-3 |
|  | Long-term potentiation | 2.2E-2 |
