# Supplementary material for: The Evolutionary Panorama of Organ-Specifically Expressed or Repressed Orthologous Genes in Nine Vertebrate Species
Source: PLoS One. 2015 Feb 13;10(2):e0116872. doi: 10.1371/journal.pone.0116872 (PMC4332667; doi:10.1371/journal.pone.0116872)
Supplement: S8 Table — (DOC) [file pone.0116872.s015.doc]

**Table S8.** DAVID functional annotation analysis of testis-specifically expressed genes.

| Category | Term | Benjamini-corrected FDR |
| --- | --- | --- |
| Go: Biological process | M phase | 1.5E-50 |
|  | cell cycle phase | 9.0E-50 |
|  | cell cycle | 1.8E-44 |
|  | cell cycle process | 3.4E-44 |
|  | M phase of mitotic cell cycle | 5.7E-35 |
|  | nuclear division | 2.7E-34 |
|  | mitosis | 2.7E-34 |
|  | organelle fission | 1.9E-33 |
|  | mitotic cell cycle | 1.6E-32 |
|  | cell division | 6.8E-28 |
|  | sexual reproduction | 1.6E-25 |
|  | spermatogenesis | 3.8E-24 |
|  | male gamete generation | 3.8E-24 |
|  | gamete generation | 6.5E-22 |
|  | reproductive process in a multicellular organism | 7.7E-18 |
|  | multicellular organism reproduction | 7.7E-18 |
|  | meiosis | 2.5E-16 |
|  | M phase of meiotic cell cycle | 2.5E-16 |
|  | meiotic cell cycle | 3.8E-16 |
| Go: Cellular component | microtubule cytoskeleton | 8.7E-43 |
|  | non-membrane-bounded organelle | 7.8E-28 |
|  | intracellular non-membrane-bounded organelle | 7.8E-28 |
|  | cytoskeletal part | 6.0E-27 |
|  | cytoskeleton | 5.9E-23 |
|  | microtubule organizing center | 6.2E-23 |
|  | spindle | 1.1E-22 |
|  | condensed chromosome | 5.5E-21 |
|  | centrosome | 5.9E-19 |
|  | microtubule | 5.9E-19 |
|  | chromosome | 1.4E-17 |
| Go: Molecular function | adenyl ribonucleotide binding | 4.4E-14 |
|  | ATP binding | 8.8E-14 |
|  | adenyl nucleotide binding | 5.9E-14 |
|  | purine nucleoside binding | 1.3E-13 |
|  | nucleoside binding | 1.6E-13 |
|  | ribonucleotide binding | 2.0E-10 |
|  | purine ribonucleotide binding | 2.0E-10 |
|  | microtubule motor activity | 2.5E-10 |
|  | purine nucleotide binding | 5.7E-10 |
|  | motor activity | 6.2E-9 |
| KEGG pathway | Cell cycle | 2.8E-11 |
|  | Oocyte meiosis | 7.2E-6 |
|  | Progesterone-mediated oocyte maturation | 2.2E-2 |
