# Supplementary material for: The Evolutionary Panorama of Organ-Specifically Expressed or Repressed Orthologous Genes in Nine Vertebrate Species
Source: PLoS One. 2015 Feb 13;10(2):e0116872. doi: 10.1371/journal.pone.0116872 (PMC4332667; doi:10.1371/journal.pone.0116872)
Supplement: S9 Table — (DOC) [file pone.0116872.s016.doc]

**Table S9.** DAVID functional annotation analysis of testis specifically-repressed genes.

| Category | Term | Benjamini-corrected FDR |
| --- | --- | --- |
| Go: Biological process | generation of precursor metabolites and energy | 1.4E-2 |
|  | cellular respiration | 1.0E-2 |
|  | energy derivation by oxidation of organic compounds | 4.5E-2 |
|  | regulation of transcription from RNA polymerase II promoter | 5.4E-2* |
|  | positive regulation of transcription | 1.4E-1* |
|  | positive regulation of gene expression | 1.4E-1* |
| Go: Cellular component | mitochondrion | 5.4E-2* |
|  | mitochondrial part | 4.9E-2 |
|  | mitochondrial membrane | 5.4E-2* |
|  | mitochondrial envelope | 5.8E-2* |
| Go: Molecular function | NAD or NADH binding | 1.5E-1* |
|  | L-malate dehydrogenase activity | 5.7E-1* |
|  | cofactor binding | 6.4E-1* |
|  | coenzyme binding | 8.2E-1* |
| KEGG pathway | Citrate cycle (TCA cycle) | 3.4E-2 |
|  | Pyruvate metabolism | 3.7E-2 |
|  | Huntington's disease | 2.6E-1* |

* Benjamini-corrected FDR is not statistically significant.
